# Influence of Hepatocellular Carcinoma on Platelet Aggregation in Cirrhosis

**DOI:** 10.3390/cancers13051150

**Published:** 2021-03-08

**Authors:** Alberto Zanetto, Marco Senzolo, Elena Campello, Cristiana Bulato, Sabrina Gavasso, Sarah Shalaby, Martina Gambato, Alessandro Vitale, Umberto Cillo, Fabio Farinati, Francesco Paolo Russo, Paolo Simioni, Patrizia Burra

**Affiliations:** 1 Gastroenterology, Department of Surgery, Oncology, and Gastroenterology, Padova University Hospital, 35128 Padova, Italy; alberto.zanetto@yahoo.it (A.Z.); marcosenzolo@hotmail.com (M.S.); sarahshalaby18@gmail.com (S.S.); fabio.farinati@unipd.it (F.F.); francescopaolo.russo@unipd.it (F.P.R.); 2 Multivisceral Transplant Unit, Department of Surgery, Oncology, and Gastroenterology, Padova University Hospital, 35128 Padova, Italy; martina.gambato@gmail.com; 3 Thrombotic and Hemorrhagic Diseases Unit, General Internal Medicine, Padova University Hospital, 35128 Padova, Italy; elena.campello@unipd.it (E.C.); cristiana.bulato@unipd.it (C.B.); sabrina.gavasso@unipd.it (S.G.); 4 Hepatobiliary Surgery and Liver Transplantation Center, Department of Surgery, Oncology, and Gastroenterology, Padova University Hospital, 35128 Padova, Italy; alessandro.vitale@unipd.it (A.V.); cillo@unipd.it (U.C.)

**Keywords:** platelets, cirrhosis, aggregation, hepatocellular carcinoma, Von Willebrand factor, cancers

## Abstract

**Simple Summary:**

Platelets are blood cells, the main function of which is to form clots and prevent/stop bleeding. However, it has been shown that platelets may be involved in additional pathophysiological processes, including stimulation of cancer growth and metastasis. In fact, inhibition of platelets in patients with various types of cancer has resulted in lower risks of cancer progression and death. This possibility has not yet been considered in patients with cirrhosis (chronic liver disease) and hepatocellular carcinoma (the most common type of liver cancer) because their platelet function has never been investigated. In this study, we show that hepatocellular carcinoma in patients with cirrhosis is associated with significantly altered (increased) platelet function. This paves the way for further studies to evaluate whether the inhibition of these hyper-functional platelets could be beneficial in patients with cirrhosis and hepatocellular carcinoma.

**Abstract:**

Hyper-functional platelets are being proposed as a potential therapeutic target in multiple cancers. Whether this can be considered in patients with cirrhosis and hepatocellular carcinoma (HCC) is unknown as their platelet function has not yet been investigated. We evaluated platelet function in cirrhosis patients with HCC. Patients with cirrhosis with and without HCC were prospectively recruited. Platelet aggregation, a marker of platelet function, was assessed by impedance aggregometry with adenosine diphosphate (ADP), arachidonic acid (ASPI), and thrombin (TRAP) stimulation. Plasmatic levels of Von Willebrand factor antigen (VWF) were also determined. One-hundred patients were recruited (50 cirrhotics with and 50 without HCC). Cirrhosis severity by Child class and platelet count were comparable between cirrhotics with and without HCC. Cirrhotics with HCC had higher ADP- (45 vs. 28; *p* < 0.001), ASPI- (47 vs. 28; *p* < 0.001), and TRAP- (85 vs. 75; *p* = 0.01) induced platelet aggregation than cirrhotics without HCC, all indicative of platelet hyper-function. The relatively increased platelet aggregation in patients with HCC was confirmed after adjusting the analysis for platelet count/severity of thrombocytopenia. Levels of VWF were higher in patients with vs. without HCC (348 vs. 267; *p* = 0.006), particularly in compensated cirrhosis. In patients with cirrhosis, HCC is associated with increased platelet aggregation and higher VWF. The clinical implications of these findings deserve further investigation.

## 1. Introduction

Platelets are increasingly recognized as important players in cancer progression and metastasis [1,2,3]. Detrimental effects of platelets in cancers include synthesis and release of growth factors normally stored within platelet granules, shielding of cancer cells from immune surveillance, facilitation of metastasis, or a combination of these factors [4,5,6,7].

On the other hand, cancer cells may release thrombopoietin and other growth factors that induce maturation of megakaryocytes and mobilization of activated platelets from the bone marrow [8].

This explains the increased platelet number and enhanced platelet function that have been demonstrated in multiple types of cancers and correlated with increased risks of thromboembolic complications and death [9,10,11].

Hepatocellular carcinoma (HCC) is the most common type of primary liver cancer and the 2nd most frequent cause of cancer-related death worldwide [12]. In approximately 90% of the cases, HCC is associated with cirrhosis [13].

Patients with cirrhosis have profound alterations of primary hemostasis that include low platelet count, increased levels of Von Willebrand factor (VWF), and complex alterations of platelet function [14,15,16,17,18,19,20], which makes platelet assessment in these patients more challenging [21].

In patients with cirrhosis and HCC, recent studies have demonstrated increased levels of platelet activation that were independently correlated with poor outcomes after treatments [22,23]. In contrast, primary hemostasis and platelet function in these patients have not yet been thoroughly investigated.

Understanding whether there is an increased platelet function in patients with cirrhosis and HCC may have significant implications because, as demonstrated for other solid cancers [10,24,25,26], the inhibition of hyper-functional platelets could potentially mitigate HCC-related morbidity and mortality [27,28].

Our goal in this prospective study was to compare platelet aggregation, a marker of platelet function, in cirrhosis patients with vs. without HCC.

## 2. Results

### 2.1. Demographics

**Figure 1:**
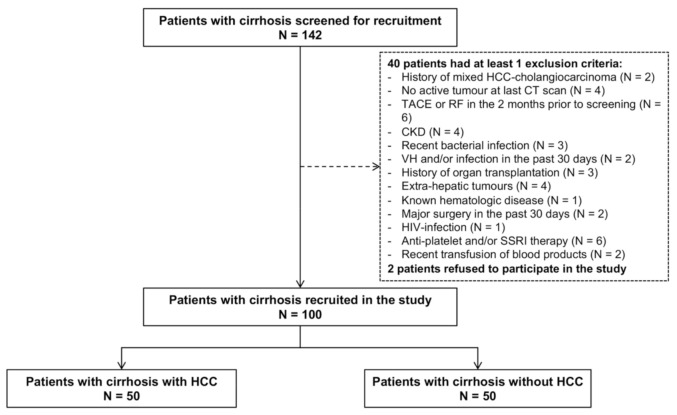
Flow-chart of the study. Legend: CKD: chronic kidney disease; VH: variceal haemorrhage; SSRI: selective serotonin reuptake inhibitors.

One hundred patients with cirrhosis were recruited (50 cirrhotics with and 50 without HCC) (Figure 1).

As shown in Table 1, baseline demographics and severity of cirrhosis were comparable between the groups. Alcohol-related was relatively more prevalent among patients with HCC whereas HCV-related cirrhosis was relatively more prevalent among patients without HCC (*p* = 0.6).

In patients with HCC, median total tumor volume (TTV) was 9 cm^3^ (5–16). Approximately 70% of patients with HCC had multinodular tumor with a median number of 3 nodules (range: 2–7). Median level of alpha-fetoprotein was significantly higher in HCC vs. non-HCC patients (9 ng/mL [4–47] vs. 3 ng/mL [2–4]). Ten patients had levels of alpha-fetoprotein ≥100 ng/mL and 4 patients ≥400 ng/mL.

Fifty per cent of patients with HCC had previously received at least 1 treatment with trans-arterial chemo-embolization and laparoscopic radiofrequency ablation being the most common types (Table 1). In treated patients, median time from last treatment for HCC to patient recruitment was 105 days (75–152 days).

### 2.2. Platelet Function in Cirrhosis Patients with vs. without HCC: HCC Is Associated with Relatively Increased Platelet Aggregation, Independent of Platelet Count

Platelet count (95 × 10^9^/L vs. 108 × 10^9^/L) and severity of thrombocytopenia were comparable between cirrhosis patients with and without HCC (Table 1).

Platelet aggregation by whole blood impedance aggregometry was positively correlated with platelet count both in healthy subjects (ADP: r = 0.3, *p* = 0.02; ASPI: r = 0.5, *p* < 0.0001; TRAP: r = 0.2; *p* = 0.08) and patients with cirrhosis (ADP: r = 0.2, *p* = 0.02; ASPI: r = 0.4, *p* < 0.0001; TRAP: r = 0.2; *p* = 0.008).

**Figure 2:**
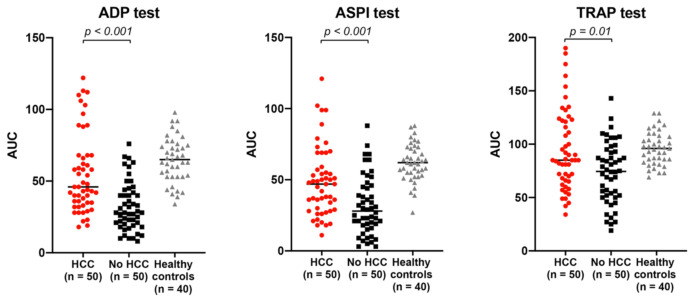
Platelet induced aggregation is significantly increased in patients with cirrhosis with vs. without HCC. Legend: HCC: hepatocellular carcinoma; ADP: adenosine diphosphate; ASPI: arachidonic acid pathway test; TRAP: thrombin receptor agonist peptide. Red circles: patients with cirrhosis with HCC. Black squares: patients with cirrhosis without HCC. Grey triangles: healthy subjects.

Platelet aggregation was significantly lower in patients with cirrhosis (both with and without HCC) than in healthy subjects with normal platelet count [234 × 10^9^/L (204–274)], independent of the agonist used (Figure 2 and Supplementary Table S1).

In patients with cirrhosis, those with HCC had significantly higher platelet aggregation than those without HCC (Figure 2). ADP-induced [45 (35–68) vs. 28 (18–43); *p* < 0.001], ASPI-induced [47 (29–62) vs. 28 (18–45); *p* < 0.001], and TRAP-induced [85 (66–121) vs. 75 (52–94); *p* = 0.01] platelet aggregation were all significantly higher in patients with vs. without HCC (Figure 2).

**Figure 3:**
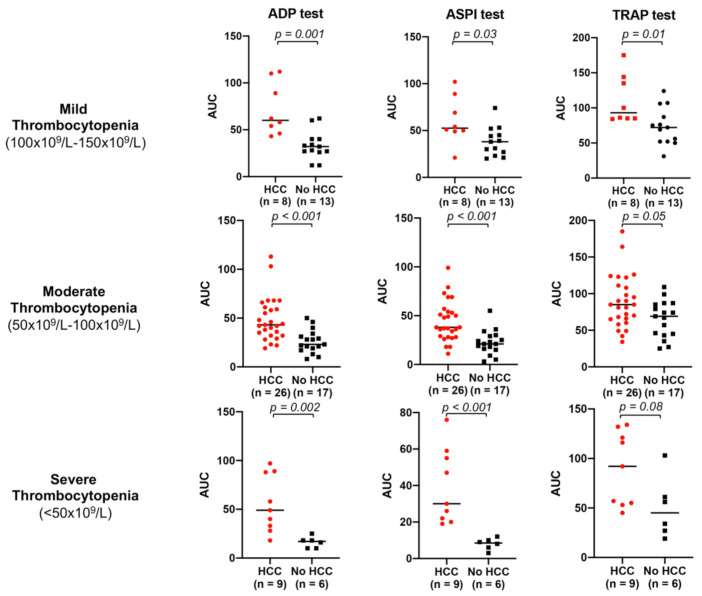
Platelet aggregation is significantly increased in cirrhosis patients with vs. without HCC in all 3 classes of thrombocytopenia. Legend: HCC: hepatocellular carcinoma; ADP: adenosine diphosphate; ASPI: arachidonic acid pathway test; TRAP: thrombin receptor agonist peptide. Red circles: patients with cirrhosis with HCC. Black squares: patients with cirrhosis without HCC. For numerical values, see Supplementary Table S2.

As the assessment of platelet aggregation by whole blood aggregometry depends on platelet count, the comparison of platelet aggregation between in cirrhosis patients with vs. without HCC was also performed according to the severity of thrombocytopenia (Figure 3). The increased platelet aggregation in cirrhosis patients with vs. without HCC was consistent with the overall findings noted above in all three subclasses of thrombocytopenia (Figure 3).

**Figure 4:**
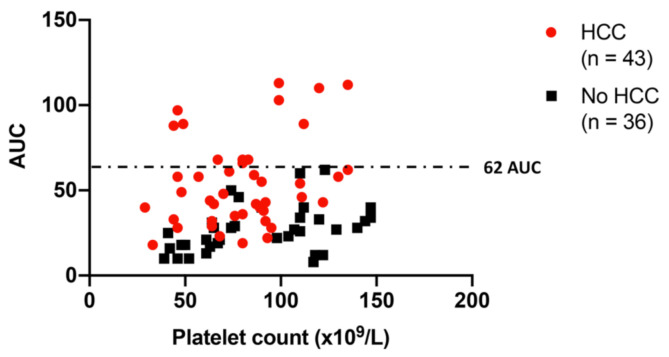
ADP-induced platelet aggregation in thrombocytopenic patients with cirrhosis with (in red) and without (in black) HCC. HCC is associated with relatively higher platelet aggregation at each level of platelet count. No patient with cirrhosis without HCC had levels of ADP-induced platelet aggregation greater than 62 AUC. Red circles: patients with cirrhosis with HCC. Black squares: patients with cirrhosis without HCC.

As shown in Figure 4, the correlation between levels of ADP-induced platelet aggregation and platelet count in thrombocytopenic patients with cirrhosis with and without HCC further demonstrates that patients with HCC had relatively higher levels of platelet aggregation compared with those without HCC at each level of platelet count (Figure 4). No patients with cirrhosis without HCC had levels of ADP-induced platelet aggregation greater than 62 AUC, independent of the thrombocytopenia severity (Figure 4).

Among patients with HCC, platelet aggregation was relatively higher in those with multinodular vs. those with a single nodule [ADP-induced: 54 AUC (36–68) vs. 40 AUC (28–56), *p* = 0.07; ASPI-induced: 51 AUC (37–69) vs. 36 AUC (21–46), *p* = 0.006; and TRAP-induced: 81 AUC (69–106) vs. 90 AUC (65–124), *p* = 0.3)], whereas no difference was found when the analysis was performed according to total tumor volume or alpha-fetoprotein level.

### 2.3. Plasmatic Marker of Primary Hemostasis in Cirrhosis Patients with vs. without HCC: HCC Is Associated with Higher Levels of Platelet Adhesive Glycoprotein Von Willebrand Factor

**Figure 5:**
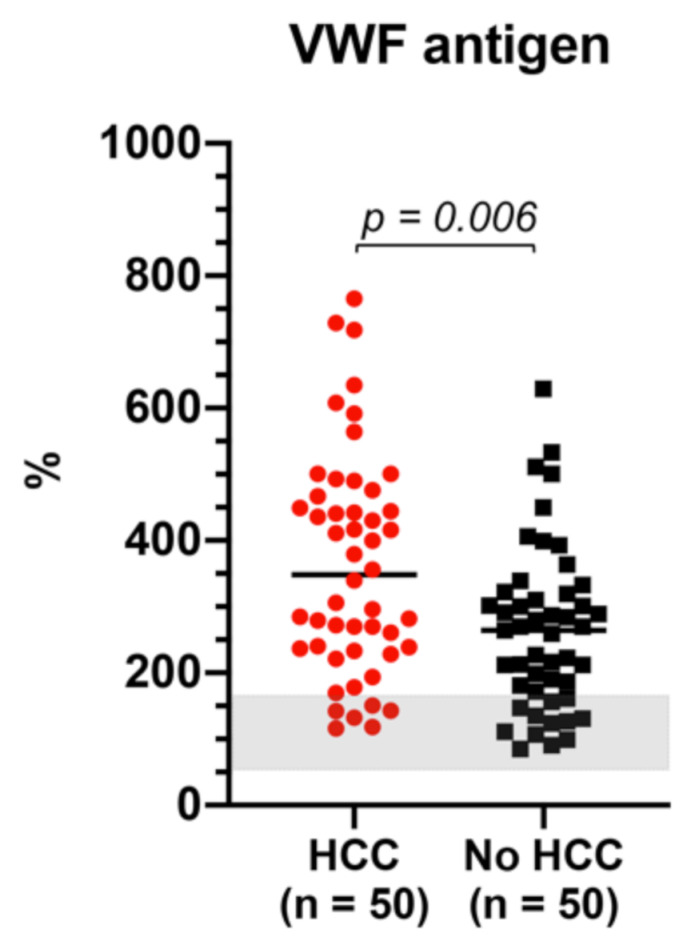
Levels of Von Willebrand factor are significantly increased in patients with vs. without HCC. Legend: HCC: hepatocellular carcinoma. Grey area refers to reference range in healthy subjects (50–160%). Red circles: patients with cirrhosis with HCC. Black squares: patients with cirrhosis without HCC.

Compared with normal reference (50–160%), patients with cirrhosis had higher levels of VWF antigen [284% (194–417)] (Figure 5).

Among patients with cirrhosis, VWF significantly increased with severity of liver disease being higher in Child C vs. Child B vs. Child A patients [380% (248–489) vs. 365% (274–448) vs. 239% (151–301), respectively; *p* < 0.0001].

Patients with cirrhosis and HCC had significantly higher levels of VWF compared with those without HCC [348% (236–469) vs. 267% (169–325); *p* = 0.006] (Figure 5).

When the analysis was adjusted according to Child class as surrogate marker of clinically significant portal hypertension, the difference of VWF levels between HCC and non-HCC patients was significant in Child A [270% (170–417) vs. 206% (138–289), *p* = 0.01] but not in Child B [423% (282–501) vs. 315% (248–401), *p* = 0.1] and Child C [436% (258–551) vs. 322% (212–450); *p* = 0.3] class.

## 3. Discussion

To our knowledge, this is the first study to demonstrate, in patients with cirrhosis, that hepatocellular carcinoma is associated with increased platelet aggregation, a marker of platelet function, independent of cirrhosis severity and platelet count. As hyper-functional platelets are increasingly recognized as a potential therapeutic target in various types of cancer [4,5,6,7], further studies are now required to assess whether inhibition of hyper-functional platelets can mitigate HCC-related morbidity and mortality in cirrhosis.

Increasing evidence suggests that platelets are important mediators in HCC carcinogenesis [26,29,30,31]. In fact, inhibition of platelet function has been correlated with reduced risks of HCC in both animal models [32] and patients with cirrhosis [27,28]. Remarkably, in a recent large retrospective analysis including 949 patients with alcohol-related cirrhosis followed for 3 years, use of aspirin was independently correlated with a significantly lower risk of HCC [33]

In patients with cirrhosis in whom HCC has already arisen, the independent association between thrombocytosis (or lack of thrombocytopenia) and increased risks of HCC recurrence and mortality is further proof of the interplay between platelets and HCC [34,35]. On one hand, HCC stimulates maturation and activation of platelets via synthesis and release of thrombopoietin, while on the other, activated platelets release multiple growth factors that stimulate growth and aggressiveness of hepatoma cells [36]. Indeed, in a recent prospective study including 191 patients with HCC who received TACE or resection and were prospectively followed after treatments, Wang et al. showed that increased levels of pre-treatment platelet activation was an independent risk factor for poor prognosis and mortality [22].

This poses the question on whether inhibition of platelet function could be considered to mitigate HCC-related morbidity and mortality. However, fear of hemorrhagic complications and lack of data regarding platelet function in patients with cirrhosis and HCC have limited the use of antiplatelet therapies in these patients.

In this study we first show that, in patients with cirrhosis, HCC is associated with increased levels of platelet aggregation, a marker of platelet function, independent of cirrhosis severity and degree of thrombocytopenia. This builds on the potential indication(s) of antiplatelet therapy in patients with cirrhosis and indicate the need for further study to address whether aspirin could be considered not only for chemo-prevention [27,28], but also to improve outcomes in cirrhosis patients in whom HCC has already arisen.

In this study, we assessed platelet aggregation by impedance whole blood aggregometry, which mimics in vivo blood conditions. However, since this assessment depends on platelet count, thrombocytopenic patients cannot be compared with those having normal platelet count [21]. In fact, previous studies on platelet function in cirrhosis have been controversial because the interpretation of platelet functional testing was challenged by the presence of thrombocytopenia [17].

To avoid this issue, we used a control group of patients with cirrhosis without HCC (with a similar severity of liver disease and a comparable platelet count), and we matched cases and controls by severity of thrombocytopenia. Remarkably, we observed that patients with HCC had relatively increased platelet aggregation not only in the overall analysis, but also at all levels of thrombocytopenia.

Interestingly, the analysis of platelet function in thrombocytopenic patients also showed that no patient with cirrhosis without HCC had levels of ADP-induced platelet aggregation higher than 62 AUC, independent of platelet count. Thus, if validated by larger studies, this threshold may become a new potential diagnostic biomarker for the diagnosis of HCC in patients with cirrhosis.

Common to all platelet stimuli is their binding to specific receptors on cell surface or the interaction with specific intra-platelet enzymes: ADP interacts with receptors P2Y1 and P2Y12, TRAP with thrombin receptor PAR-1, and arachidonic acid (ASPI) with intra-platelet cyclooxygenase-1. In our cohort, platelet aggregation was significantly higher in patients with HCC, independent of the agonist used. This suggests that HCC does not interfere with a single pathway, but probably acts downstream in the activation/aggregation process.

The main hemostatic functions of Von Willebrand factor, a glycoprotein released by endothelial cells, are to carry FVIII and facilitate the interaction between platelets and sub-endothelial collagen [37,38]. However, in addition to its hemostatic properties, recent evidence suggests that VWF may have additional role(s) in modulating cancer cells biology [39]. It has been shown that cancer cells, including those of non-endothelial origin, may acquire the ability to synthesize and release VWF, which then facilitates cancer progression and metastasis [40,41,42]. In fact, plasmatic levels of VWF have been proposed as prognostic markers in patients with solid cancer [43,44,45,46], including those with HCC, in whom high levels of VWF have been correlated with increased rates of tumor recurrence after treatments [47].

Similarly to what was previously described in other types of solid tumors [48], we found plasmatic levels of VWF to be significantly higher in HCC vs. non-HCC patients. However, when patients were matched according to Child class as surrogate marker of clinically significant portal hypertension, the difference between HCC and non-HCC groups was more profound in compensated (Child A) rather than in decompensated (Child B and C) patients. This suggests that, in patients with more advanced chronic liver disease, the HCC-driven effect on Von Willebrand factor is overridden by the underlying endothelial shear stress due to portal hypertension. However, as we included determination of VWF antigen alone, more comprehensive studies including VWF active form and/or levels of VWF propeptide are required to confirm these findings and more properly evaluate the effect of HCC on VWF axis [49,50,51]. Therefore, whether levels of active VWF are truly increased in cirrhosis patients with HCC, and whether increased levels of VWF could have prognostic utility or become an additional therapeutic target, particularly in compensated cirrhosis, should be further explored.

As our main objective was the assessment of platelet aggregation, we did not include evaluation of endothelial function. However, recent data suggest that alterations of endothelial function may also be involved in cancer development and progression [52,53]. In patients with cirrhosis, endothelial dysfunction may have a role in development of complications and disease progression [54,55]. Specifically, in cirrhosis with HCC, we previously demonstrated that levels of plasmatic endothelium-derived microvesicles are increased in patients with vs. without HCC, thus suggesting that HCC may be associated with endothelial activation in these patients [56]. Future study should evaluate more specific endothelial cell inflammatory markers (i.e., soluble *p*-selectin) in cirrhosis with HCC and assess whether such markers could have prognostic or therapeutic potential in these patients.

In the only previous study that looked at platelet activation status and levels of VWF in cirrhosis patients with and without HCC, Alkozai et al. did not find any difference between groups [57]. However, compared with our cohort, patients with HCC had smaller tumors with fewer nodules and no patients had evidence of extra-hepatic metastasis [57]. It has been suggested that tumor characteristics may have a role in determining changes in primary hemostasis in patients with cirrhosis [58]. Our finding that patients with a multinodular tumor had a relatively higher platelet aggregation than those with a single nodule would indicate that the effect of HCC on primary hemostasis may be different in less vs. more aggressive/advanced HCC. To this end, in a recent large comprehensive study including 100 patients with HCC, Zhang et al. showed not only that platelet activation status, as assessed by percentage of *p*-selective+ platelets at cytofluorimetry, was increased in patients with HCC compared with healthy subjects, but also that patients with poorly differentiated HCC had increased platelet activation compared with those with moderate or well differentiated HCC, which demonstrates that HCC characteristics may influence the degree of platelet activation [23]. However, as the number of patients with HCC included in our study did not allow a comprehensive analysis to be performed of platelet aggregation according to tumor characteristics, and as we were unable to find any difference according to other HCC characteristics such as tumor volume or levels of alfa-fetoprotein, larger studies are required to validate this hypothesis and to further look at the correlation between alterations of platelet function and HCC characteristics.

Our study has some limitations. First, as in any in vitro study regarding hemostasis, the loss of interplay between blood components and vessel walls is unavoidable. However, whole blood impedance aggregometry is a methodology that provides an assay milieu that mimics the in vivo condition. In fact, it is performed in whole blood, thus enabling other blood elements to influence platelet aggregation, and takes place on a solid surface, thus somewhat resembling the physiological process of platelet adhesion and aggregation. Drugs could also have interfered with platelets, but this effect should have been mitigated by use of the same inclusion criteria for both groups and the exclusion of other confounders such as renal dysfunction, bleeding and thrombosis, extra-hepatic cancers, and use of antiplatelet therapy and selective-serotonin uptake inhibitors. Second, other potential parameters correlated with platelet and endothelial function such as mean platelet volume, platelet distribution width, levels of VWF active form/propeptide, and levels of *p*-selectin were not included in this study. Finally, as we aimed to investigate alterations of primary hemostasis, important HCC-driven changes in coagulation and fibrinolysis were not described.

In conclusion, in a prospective study in patients with cirrhosis, we demonstrate that hepatocellular carcinoma is associated with significant changes in primary hemostasis that include increased platelet aggregation and higher levels of plasmatic von Willebrand factor. Further studies are required to evaluate whether these alterations could have prognostic utility or whether they can become new therapeutic targets to mitigate HCC-related morbidity and mortality in patients with cirrhosis.

## 4. Materials and Methods

### 4.1. Patient Selection

Adult (>18 years old) patients with cirrhosis who attended or were referred to the liver clinics of Gastroenterology, Multivisceral Transplant Unit, and Hepatobiliary Surgery and Liver Transplantation Center of Padova University Hospital from 1 July 2020 to 30 October 2020 were prospectively screened to determine eligibility to participate in the study.

The diagnosis of cirrhosis was confirmed with available data including histology, radiology, laboratory and clinical assessment. Decompensation was defined by the presence or history of clinically evident decompensating events (ascites, variceal hemorrhage, and hepatic encephalopathy) [59,60]. Diagnosis of HCC was based on the European Association for the Study of the Liver clinical practice guidelines [12].

Patients with a history of HCC and no evidence of active tumor at the last available imaging, patients with active HCC but who had a treatment-free interval of less than 2 months at time of screening, and patients with a history of mixed HCC-cholangiocarcinoma were not eligible.

At screening, patient’s medical records, past medical history, and laboratory data were reviewed for the following exclusion criteria: history of portal hypertensive-related bleeding and/or any other major bleeding [61] and/or any bacterial infection in the 30 days prior to evaluation; acute and chronic kidney disease; presence or history of extra-hepatic tumors or known hematologic diseases; recent surgery (within 30 days); HIV-infection, history of any organ transplantation, including liver; antiplatelet and/or anticoagulant therapy; treatment with any selective serotonin reuptake inhibitors; and recent (1 week) transfusion of any blood product.

Age and sex-matched healthy subjects were recruited as controls for platelet function. This group constituted 40 healthy subjects with normal platelet count, no history of acute or chronic disease. None of the controls were taking antithrombotic, anticoagulant, antibiotic, or hormonal therapy.

### 4.2. Study Design

This was a prospective, single-center, cohort study, approved by the Padova University Hospital Ethical Committee (protocol #0034435-08/06/20). The study was conducted in compliance with the Declaration of Helsinki and all patients gave written informed consent before enrollment.

### 4.3. Sample Collection and Primary Hemostasis Assessment

#### 4.3.1. Blood Sampling

Peripheral blood was collected via venipuncture in citrate-containing vacutainer tubes using 21 g needles and a tourniquet. Platelet-poor plasma was prepared within 1 h by double centrifugation (2 × 10 min at 1500 g) at room temperature. Aliquots (1 mL) were immediately frozen and then stored at −80 °C until use.

#### 4.3.2. Platelet Function Assessment

In vivo, when sub-endothelial matrix is directly exposed to blood flow, platelets adhere directly to collagen or indirectly via the interposition of platelet adhesive glycoprotein VWF. Adhesion leads to platelet activation and aggregation.

In vitro, platelet aggregation can be measured by impedance whole blood aggregometry (Multiplate^®^ function analyzer, Roche Diagnostics, Rotkreuz, Switzerland), which provides an assay milieu that mimics in vivo platelet activation and aggregation. Multiplate^®^ was performed within 2 h after blood draw by one trained member of the research team (AZ), as previously reported [62,63,64].

Evaluation of platelet aggregation by Multiplate^®^ is based on the impedance method. The analysis takes place in a single-use test cell, which incorporates dual copper sensor wires. Upon activation by different agonists, platelets adhere to the sensor wires and thereby increase the electrical resistance (i.e., impedance). The increase is proportional to the capability of platelets to aggregate on each wire. Results are expressed as Area Under the Curve (AUC, AU*min). The higher the AUC value, the greater the capability of platelets to aggregate.

Per our protocol, platelets were stimulated with 3 different agonists: (1) ADP 6.5 umol/L (ADP test—Roche Diagnostics GmbH, Mannheim, Germany); (2) arachidonic acid 500 umol/L, which allows the evaluation of cyclooxygenase-dependent aggregation (ASPI test—Roche Diagnostics GmbH, Mannheim, Germany); (3) thrombin receptor activating peptide-6 (TRAP-6) 32 umol/L, which is the most potent platelet activator and stimulates platelet aggregation via the thrombin receptor PAR-1 (TRAP test—Roche Diagnostics GmbH, Mannheim, Germany).

#### 4.3.3. Von Willebrand Factor

Von Willebrand factor (VWF) is a plasmatic glycoprotein released by endothelial cells, which facilitates the interaction between platelets and sub-endothelial collagen at the site of vascular injury. In patients with cirrhosis, VWF is increased due to portal hypertension and endothelial shear stress [65,66]. Because VWF plays a role in modulating platelet function, especially in patients with cirrhosis [67], and increased levels of VWF have been previously described in patients with other types of solid cancers [48], we also evaluated determination of VWF in all patients with cirrhosis.

Per our protocol, levels of VWF antigen (VWF:Ag) were determined by latex immunoturbidimetric assay (STA^®^-Liatest^®^VWF:Ag, Diagnostica Stago, Asnieres-Sur-Seine, France) in sodium citrated plasma using the automated STA Compact Max^®^ coagulation analyzer (Diagnostica Stago) according to manufacturer’s instructions.

### 4.4. Data Collection

Data collected from the medical record included patient demographics, etiology and severity of cirrhosis, and laboratory data.

Model for End-Stage Liver Disease score and Child class were calculated based on biochemical values and clinical characteristics from the day of enrollment.

Thrombocytopenia was defined by a platelet count ≤150 × 10^9^/L and sub-classified as mild (range: 100 × 10^9^/L–150 × 10^9^/L), moderate (range: 50 × 10^9^/L–<100 × 10^9^/L) or severe (range: <50 × 10^9^/L) [68].

In patients with HCC, the following variables were also collected: number of nodules, size of each nodule, presence of neoplastic thrombosis or extra-hepatic spread, history of previous treatment for the HCC, and HCC stage according to the Barcelona Clinic Liver Cancer staging [12].

Total tumor volume (TTV) was calculated as the sum of the volumes of all tumors according to the following formula: (4/3) π r3, where r is the maximum radius of each nodule of HCC [69].

### 4.5. Data Analysis

#### 4.5.1. Sample Size Determination

In our previous study regarding hemostatic alterations in patients with cirrhosis and HCC, these patients had a significantly higher platelet count compared with patients without HCC (122 × 10^9^/L vs. 87 × 10^9^/L) [58]. Since when designing this protocol we did not find any study regarding alterations of platelet function in cirrhosis patients with HCC, we based our sample size on that difference. Sample size was calculated assuming a continuous endpoint compared between two independent groups, two-sided type I error of 0.05, and statistical power of 0.90. The required per-group sample size was 48.

#### 4.5.2. Statistical Analysis

Qualitative data are described using frequency and percentage. Quantitative data are described using median with 25% and 75% quartile ranges. Comparison between independent groups were performed using the Mann Whitney U test and *t*-test for continuous variables, and Chi-square test of Fisher’s exact test for categorical variables. Statistical significance was set at *p* ≤ 0.05. All analyses were completed using SAS version 9.4 (SAS Institute Inc., Cary, NC, USA).

## 5. Conclusions

Hepatocellular carcinoma in patients with cirrhosis is associated with increased platelet aggregation and higher levels of platelet adhesive glycoprotein von Willebrand factor.

Further studies are required to evaluate whether these alterations could have prognostic utility or could become new therapeutic targets to mitigate HCC-related morbidity and mortality in patients with cirrhosis.

## Figures and Tables

**Table 1:** Baseline characteristics in patients with cirrhosis.

Variables	HCC (*n* = 50)	No HCC (*n* = 50)
Age, years	65 (58–69)	61 (55–71)
Male gender, %	80	66
Etiology of cirrhosis, %		
Alcohol	46	36
HCV	26	42
NASH	10	8
HBV ± HDV	16	12
Other	2	2
Child class A/B/C, %	46/36/18	62/22/16
MELD score	11 (8–16)	10 (8–14)
History of decompensation, %		
Ascites	46	28
Variceal hemorrhage	22	22
Hepatic encephalopathy	6	20
Diabetes, %	28	32
Hemoglobin, g/dL	12 (11–14)	12 (10–14)
Platelet count, 10^9^/L	95 (64–114)	108 (67–140)
Thrombocytopenia, (%)		
Present	86	72
Mild 100–150 × 10^9^/L	19	33
Moderate 50–100 × 10^9^/L	60	50
Severe <50 × 10^9^/L	21	17
Total bilirubin, mg/dL	1.2 (0.9–2.7)	1.2 (0.8–3.5)
INR	1.3 (1.2–1.5)	1.2 (1.1–1.6)
Creatinine, mg/dL	0.8 (0.7–0.9)	0.8 (0.7–0.9)
Albumin, g/dL	31 (29–36)	34 (29–38)
AFP, ng/mL	9 (4–47)	3 (2–4)
Multinodular, %	68	-
Number of nodules	3 (2–7)	-
TTV, cm^3^	9 (5–16)	-
TTV > 10 cm^3^, %	45	-
History of previous treatment, %		-
Yes/No	45	
TACE ^§^	39 in 19	
RF ^§^	31 in 20	
Resection ^§^	5 in 5	
PEI	2 in 2	
Capecitabin	1	
BCLC staging 0/A/B/C/D, %	10/19/57/8/6	-

Median values reported with 25th and 75th percentile values in parenthesis. Abbreviations: HCV: hepatitis C virus; NASH: non-alcoholic steatohepatitis; HBV: hepatitis B virus; HDV: hepatitis D virus; MELD: Model for End-Stage Liver Disease; AFP: alpha-fetoprotein; TTV: total tumor volume; TACE: trans-arterial chemoembolization; RF: radiofrequency; PEI: percutaneous ethanol injection; BCLC: Barcelona Clinic Liver Cancer staging. ^§^ Number of procedures in n patients.

## Data Availability

The authors confirm that the data supporting the findings of this study are available within the article and its supplementary materials.

## Supplementary Materials

The following are available online at https://www.mdpi.com/2072-6694/13/5/1150/s1, Table S1: Platelet aggregation (AUC) in patients with cirrhosis vs. healthy subjects; Table S2: Platelet aggregation (AUC) in patients with cirrhosis with vs. without HCC according to severity of thrombocytopenia.
