# Supplementary material for: Influence of Hepatocellular Carcinoma on Platelet Aggregation in Cirrhosis
Source: Cancers (Basel). 2021 Mar 8;13(5):1150. doi: 10.3390/cancers13051150 (PMC7962527; doi:10.3390/cancers13051150)
Supplement: Supplementary file 1 [file cancers-13-01150-s001.pdf]

Supplementary Table 1. Platelet aggregation (AUC) in patients with cirrhosis vs. healthy subjects

	**Patients with cirrhosis with HCC (n=50)**	**Patients with cirrhosis without HCC (n=50)**	**Healthy subjects (n=40)**
**ADP**	45 (35-68)	28 (18-43)	63 (53-74)
**ASPI**	47 (29-62)	28 (18-45)	62 (55-72)
**TRAP**	85 (66-121)	75 (52-94)	96 (86-107)

Median values reported with 25th and 75th percentile values in parenthesis. Abbreviations: ADP: adenosine diphosphate; ASPI: arachidonic acid test; TRAP: thrombin receptor activated peptide;

Supplementary Table 2. Platelet aggregation (AUC) in patients with cirrhosis with vs. without HCC according to severity of thrombocytopenia

	**HCC**	**No HCC**
**Mild thrombocytopenia**	(n=8)	(n=13)
**ADP**	60 (48-105)	32 (27-40)
**ASPI**	53 (49-84)	38 (25-89)
**TRAP**	93 (85-142)	72 (52-97)
**Moderate thrombocytopenia**	(n=26)	(n=17)
**ADP**	43 (32-62)	23 (18-33)
**ASPI**	38 (28-55)	21 (16-30)
**TRAP**	83 (64-109)	69 (44-86)
**Severe thrombocytopenia**	(n=9)	(n=6)
**ADP**	49 (31-89)	17 (10-20)
**ASPI**	30 (21-57)	9 (5-11)
**TRAP**	92 (54-127)	45 (25-72)

Median values reported with 25th and 75th percentile values in parenthesis. Abbreviations: ADP: adenosine diphosphate; ASPI: arachidonic acid test; TRAP: thrombin receptor activated peptide;
